# Reversed Procrastination by Focal Disruption of Medial Frontal Cortex

**DOI:** 10.1016/j.cub.2016.08.016

**Published:** 2016-11-07

**Authors:** Ashwani Jha, Beate Diehl, Catherine Scott, Andrew W. McEvoy, Parashkev Nachev

**Affiliations:** 1 Institute of Neurology, UCL, Queen Square, London WC1N 3BG, UK; 2 National Hospital for Neurology and Neurosurgery, Queen Square, London WC1N 3BG, UK

**Keywords:** pre-supplementary motor area, voluntary action, direct cortical stimulation, race models of decision-making, brain image registration

## Abstract

An enduring puzzle in the neuroscience of voluntary action is the origin of the remarkably wide dispersion of the reaction time distribution, an interval far greater than is explained by synaptic or signal transductive noise [1, 2]. That we are able to change our planned actions—a key criterion of volition [3]—so close to the time of their onset implies decision-making must reach deep into the execution of action itself [4, 5, 6]. It has been influentially suggested the reaction time distribution therefore reflects deliberate neural procrastination [7], giving alternative response tendencies sufficient time for fair competition in pursuing a decision threshold that determines which one is behaviorally manifest: a race model, where action selection and execution are closely interrelated [8, 9, 10, 11]. Although the medial frontal cortex exhibits a sensitivity to reaction time on functional imaging that is consistent with such a mechanism [12, 13, 14], direct evidence from disruptive studies has hitherto been lacking. If movement-generating and movement-delaying neural substrates are closely co-localized here, a large-scale lesion will inevitably mask any acceleration, for the movement itself could be disrupted. Circumventing this problem, here we observed focal intracranial electrical disruption of the medial frontal wall in the context of the pre-surgical evaluation of two patients with epilepsy temporarily reversing such hypothesized procrastination. Effector-specific behavioral acceleration, time-locked to the period of electrical disruption, occurred exclusively at a specific locus at the ventral border of the pre-supplementary motor area. A cardinal prediction of race models of voluntary action is thereby substantiated in the human brain.

## Highlights

• Voluntary reaction times are slower and more variable than neural noise explains
• Such procrastination is theorized to reflect a neural race selecting each action
• Electrically disrupting medial frontal cortex reverses procrastination
• A cardinal prediction of race models of action in the brain is thereby confirmed

## Teaser

Studying the effects of direct cortical stimulation of the human medial frontal lobe, Jha et al. report reversal of the natural action delay or “procrastination” long predicted by race models of voluntary action as a fundamental feature of decision-making in the human brain.

## Results and Discussion

The transient, focal, “virtual” lesioning effected by intracerebral electrical stimulation of cortex for clinical purposes opens a rare but illuminating window into the operations of the human cortex [15, 16]. Though stringently constrained, behaviorally and anatomically, to the actions and neural loci justified by clinical need, in the two patients studied here the clinical requirements of evaluation for possible epilepsy surgery serendipitously overlapped with the present scientific question. Subdural electrode grids were temporarily implanted on the medial surface, among other areas, of the left frontal lobe, with the aim of identifying a presumed epileptic focus whose location anatomical neuroimaging had not disclosed and for mapping motor areas on the medial wall should a resection there be subsequently indicated (see Supplemental Experimental Procedures and Table S1).

Following standard clinical practice, the patients were asked to perform self-paced, repetitive actions—vocally or manually in different blocks—while electrical current was briefly delivered between pairs of neighboring grid electrodes, one pair at a time, for manually controlled durations of a few seconds [17]. Each action consisted of alternating movements at a frequency the patient chose spontaneously but was asked to maintain at a constant value. In the manual task, the movements were single or multiple finger flexions and extensions at the proximal metacarpophalangeal joints; in the vocal task, the movements were repeated single syllable vocalizations such as “la-la-la” (see Movie S1). Patients were also tested at rest and during natural reading. The electrical stimulation parameters—50 Hz biphasic square wave delivering up to 4 mA of current between two adjacent electrodes 5 mm apart—were within the values generally considered disruptive of underlying neural function [16, 18]. Since cortical stimulation may readily trigger seizure activity, clinical practice constrained behavioral testing to no more than one or two blocks per electrode contact and target behavior.

To establish the underlying anatomy, the electrode locations imaged with computed tomography (CT) were co-registered to a pre-implantation volumetric magnetic resonance (MR) volume, correcting for post-operative brain distortion, with independent manual landmark validation of the result. The images were then non-linearly transformed into standard stereotactic anatomical space (Montreal Neurological Institute [MNI]), guided by the MR image. Individual motor anatomical context was provided by fMRI of noun repetition and verb generation: this highlighted the individual location of the medial motor areas. Each electrode contact on the medial wall was thus localized by individual structural anatomy, MNI template coordinates, and functional markers of speech articulation and generation (Figure 2; see Supplemental Experimental Procedures).

**Figure 1:**
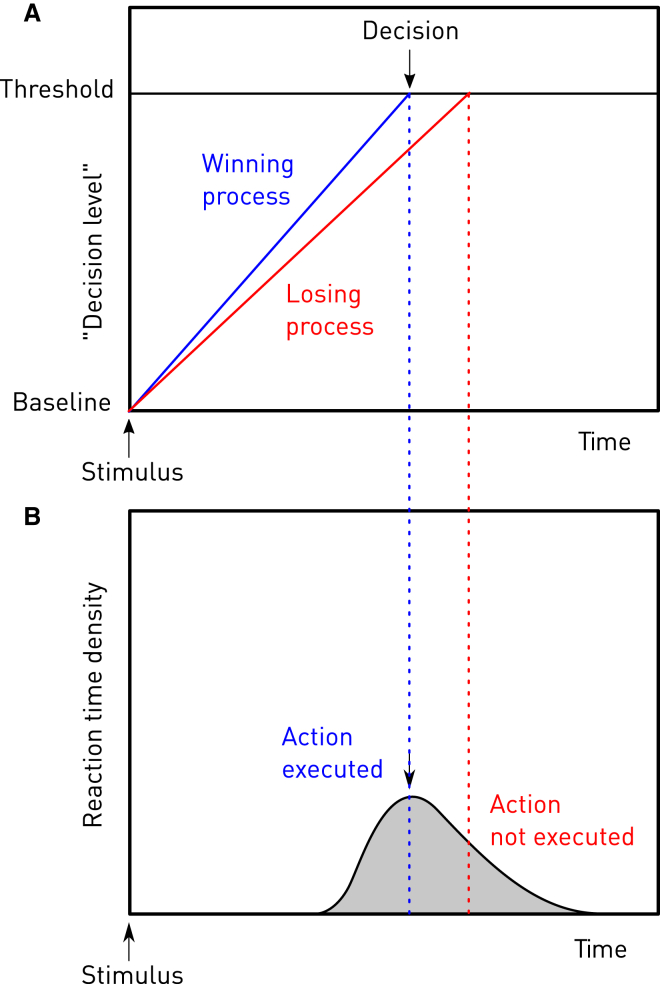
Decision-Making as a Race (A and B) Race models of voluntary action conceive of an ensemble of decision signals embodying a measure of the probability of an action that rise linearly from baseline, each at a given rate, to approach a critical threshold (A). The action executed on any given occasion corresponds to that associated with the signal reaching the threshold first (in blue). Variation in the winner on any one occasion, resulting from variability in the race parameters, generates the characteristic distribution in reaction times (B). Although only two processes are shown here, a multiplicity of processes will compete for the threshold at any one time, reflective of the wide horizon of action possibilities before us. Within the LATER race model employed here, the decision process is conceptualized as a log measure of the probability of the corresponding action. Note that the start of the race is commonly timed by an external stimulus event, but the same principle may apply to any condition relevant to action, including internal physiological states.

The combination of self-pacing and alternating between two isolable actions—finger flexion and extension versus pausing, or vocalization versus glottal arrest—offered as optimal a test of neural procrastination as a coincidence with clinical protocol could be expected to yield. Within the family of race models of action, an ensemble of decision signals embodying a measure of the probability of an action rise linearly from baseline, at a given rate, to approach a critical threshold (Figure 1). The action executed on any given occasion corresponds to the signal reaching the threshold first. Whereas in exogenously driven action, the source of the decision signals is the external environment—visual cues, for example—in endogenously driven, self-paced action, the source might only be internal states reflecting desired goals. And whereas the race outcome may be manifest in the *morphology* of the response—for example, moving a finger left versus right—in self-paced, identically repetitive action, it may be manifest only in the *timing* of the response—for example, low versus high repetition rates. Now, if the race distance is artificially shortened, by either raising the baseline or lowering the threshold, early completion of the race underlying each action will result. In the context of repetitive, self-paced behavior, reversed neural procrastination thus predicts an increase in the overall frequency, whatever the subject’s desired pace of alternation.

**Figure 2:**
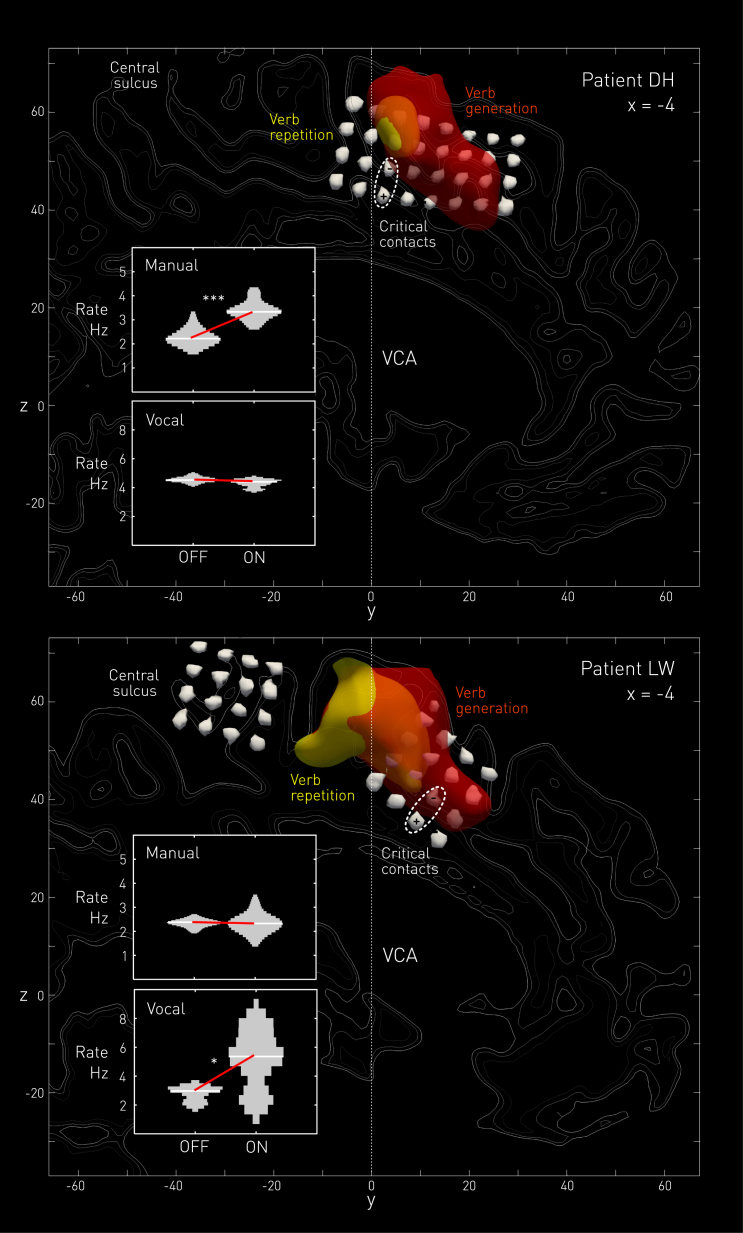
Structural and Functional Localization of Stimulation Sites on the Medial Frontal Wall and Associated Behavior In separate panels for each patient are shown renders of the MR structural, MR functional, and CT post-electrode-implantation imaging, all non-linearly transformed into standard MNI stereotactic space by a unified normalization and segmentation procedure implemented in SPM12. For each patient, the MR structural image (a pre-implantation T1-weighted 0.94 × 0.94 × 1.1 mm acquisition from which the MNI normalization parameters were derived for all other imaging) is represented for clarity by the estimated gray matter compartment only, with isolines corresponding to the 90%, 80%, and 70% probability contours, in that order of increasing intensity, cut through a parasagittal plane at x = −4 mm. The functional imaging data, performed before implantation and derived from blocked verb repetition (yellow) or verb generation (red) compared with rest, were used to compute SPM *t*-statistic maps of significant task-related activation, which were then rigidly co-registered to the structural scan via the mean echoplanar image and subsequently transformed into MNI space. Semi-transparent contours of the clusters on the medial wall are thresholded at p = 0.05 family-wise error corrected, except for verb generation in DH where weak activation necessitated a drop in threshold to p = 0.001 uncorrected. The CT post-electrode-implantation image, a 0.43 × 0.43 × 1.2 mm acquisition, was rigidly co-registered to the pre-operative MR volume and then non-linearly adjusted by a unified normalization and segmentation procedure with the previously estimated, smoothed native space MR tissue compartments applied as priors. The non-linear adjustment was applied to compensate for the subtle but noticeable descent of the dorsal surface following craniotomy so as to improve the accuracy of contact localization in the dorsoventral plane. As with the others, this adjusted image was then transformed into MNI space using the parameters derived from the MR image, resampled to 1 × 1 × 1 mm resolution. Each grid contact was then visualized by rendering with a contour thresholded at metal density, within a region of interest enclosing the medial wall so as to exclude both bone and contacts elsewhere in the brain. The critical loci where a behavioral effect was observed are enclosed by dashed ellipses, lying on the ventral border of the pre-supplementary motor area. Note that since the stimulation current was biphasic, the polarity of the electrodes reversed at 50 Hz. The insets show violin plots of the distributions of the reciprocals of the inter-movement intervals—essentially instantaneous frequency, in Hz—for the alternating tasks the patients performed, both manually and vocally, while the critical contacts were stimulated. The manual task consisted of self-paced, repetitive finger flexion and extension movements; the vocal task consisted of equally self-paced, repetitive single syllable vocalizations of the form “la-la-la.” The red lines index the change in the locations of the distributions, showing a significant increase in behavioral frequency in the manual task for DH (p < 0.001, Bonferroni adjusted, marked ^∗∗∗^) and in the vocal task for LW (p = 0.030, Bonferroni adjusted, marked ^∗^), consistent with effector-specific inhibition of procrastination. See also Figures S1–S3, Tables S1 and S2, and Movie S1.

This is precisely what we observed in each patient, at a location falling within the ventral pre-supplementary motor area on the border between the superior frontal gyrus and the cingulate gyrus, inside 10 mm rostral of the VCA line (Figure 2). Disruptive stimulation here, and only here among all electrode contacts, visibly and audibly increased the frequency of alternation, leaving the morphology of the movement otherwise unchanged (see Movie S1 and Table S2). This inference was formalized by comparing the distributions of inter-movement intervals in blocks immediately before and after stimulation onset, as determined from video telemetry and audio spectral analysis, within a mixed general linear model with stimulation and effector as fixed factors and patient as a random factor (see Supplemental Experimental Procedures). There was a significant main effect of stimulation consisting of an increase in behavioral frequency from a mean of 2.96 Hz (SEM = 0.35) to 3.76 Hz (SEM = 0.34) (*F(1,123)* = 22.547, p < 0.001). The behavioral acceleration was strongly effector specific: in the patient where the electrodes were slightly more rostral, LW, the acceleration was confined to vocalization; in DH, the converse was observed (Figure 2, inset plots, and Table S2). This was reflected in a highly significant three-way interaction of stimulation, patient, and effector (*F(4,123)* = 9.547, p < 0.001), with post hoc t tests confirming in LW a vocal (p = 0.030), but not manual, effect (p = 1.000), and in DH a manual (p < 0.001), but not vocal, effect (p = 0.098, all Bonferroni adjusted).

**Figure 3:**
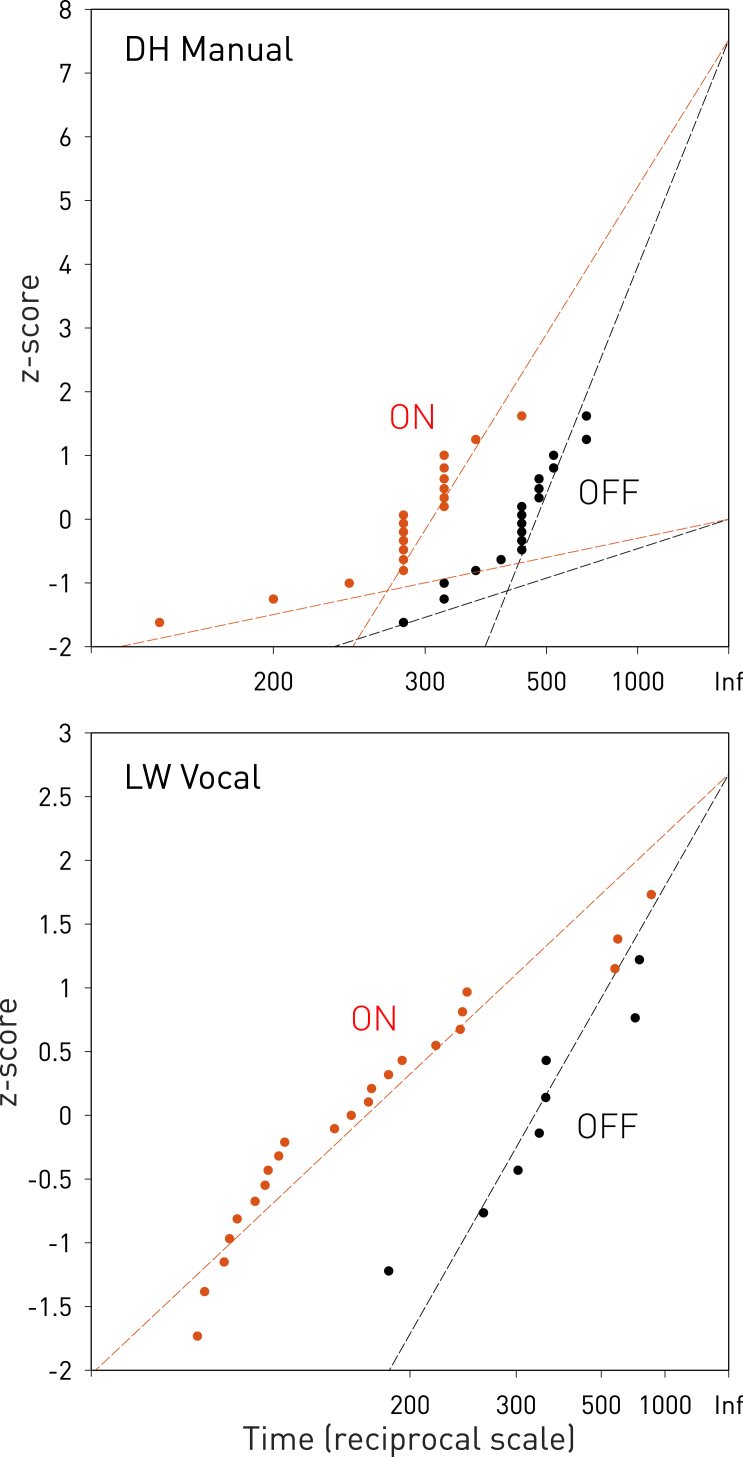
LATER Analysis of Inter-movement Intervals Although the patients made self-paced alternating movements, it is licit to treat the inter-movement intervals as reaction times relative to an endogenous timing signal setting the individual rate of alternation. The observation catalytic of the LATER model—that reaction times show a linear relationship when plotted as their reciprocals against their cumulative (assumed Gaussian) distribution—can thus be tested on our data. Plotted here so transformed are the intervals for the electrodes where a significant effect of stimulation (in red) was observed (the manual task in DH, top, and the vocal task in LW, bottom) with time on a reciprocal scale as the abscissa and the *Z* score as the ordinate index of position within a Gaussian distribution. Maximum likelihood fits of the major components of the distributions and (only in DH where it was present) separately for the minor early components are given in dashed lines. According to the LATER model, stimulation-induced reversed procrastination predicts a change in the slope of the function, causing it to swivel around a fixed intercept, whereas acceleration of the competing processes predicts a shift to the left along the abscissa, leaving the slope unchanged. Model comparison using the BIC as the metric of modeling felicity indicated that swivel was better than shift (change in BIC = 4.82, substantial evidence). It was also better than both the unconstrained (change in BIC = 13.45, very strong evidence) and the null model (change in BIC = 32.38, very strong evidence). LATER analysis thus here supports reversed procrastination. See Supplemental Experimental Procedures for details. Note the discretization of timing data in DH is a consequence of the relatively sparse temporal sampling of standard clinical video recording (every 40 ms). LATER modeling was performed using Mike Shadlen’s Reciprobit Toolbox v.1.0. See also Figures S1 and S2.

To illuminate the underlying mechanisms, we further modeled the inter-movement intervals as if they were natural reaction times [19, 20], reasonably assuming the timing of each component movement to be relative to endogenous determinants of the self-paced rate of alternation. The duration of each component movement was generally shorter than the inter-movement interval, making such discretization mechanistically plausible. This enabled us to perform a reciprobit analysis where the intervals were transformed to their reciprocals and plotted against their cumulative distribution, on the assumption it is Gaussian after the transformation. The resultant distributions could be adequately modeled by linear functions, demonstrating that a race model, specifically the standard LATER (linear approach to threshold with ergodic rate) model, fits the observed behavior [8, 21] (Figure 3).

Moreover, the change in the underlying function produced by stimulation was exactly as the LATER model would predict if the “decision distance” from baseline to threshold were diminished, i.e., if the race were artificially shortened. In such circumstances, LATER predicts a “swivel” of one function against the other around a fixed intercept at infinity, whereas if the rate of rise of the neural processes were increased, there should be a “shift” along the time axis, the slope of the function remaining unchanged. To determine which of these two alternatives best agreed with the data, we fitted LATER models where either the intercept (swivel model) or the slope (shift model) was fixed across the stimulation factor. We also estimated an unconstrained model, where each of these parameters was allowed to vary, and a null model, where none could. Model comparison using the Bayesian information criterion (BIC) as the metric of modeling felicity indicated that swivel was better than shift (change in BIC = 4.82, substantial evidence). It was also better than both the unconstrained (change in BIC = 13.45, very strong evidence) and the null model (change in BIC = 32.38, very strong evidence). LATER not only fits the observed natural behavior here, reversed procrastination is the effect of stimulation it favors over other alternatives. Parameter estimates from the winning model indicated a relative reduction in the decision threshold by a factor of 0.65 (bootstrapped confidence intervals [CI] 0.58 to 0.78) in the motor condition and a factor of 0.56 (bootstrapped CI 0.41 to 0.72) in the vocal condition.

All race models of action naturally allow for effector specificity, for two or more action possibilities may be said to compete only to the extent to which they share an effector. The dissociation we observed may be reflective of the underlying rostrocaudal somatotopic organization of the medial wall, though the functional boundaries of vocalization-sensitive areas on the medial wall independently determined by individual fMRI as part of the clinical investigation of the patient are comparatively wide (Figure 2, red and yellow plots). The most rostral boundary of the cortex significantly activated by verb generation is clearly closer in LW than in DH to the critical stimulation contacts, but this is difficult to interpret given the smoothness of the functional data and inter-individual differences here in the task-specific BOLD signal.

What effector specificity does demonstrate, however, is that the reversed procrastination effect cannot plausibly result from a global confound such as arousal, nor from remote effects such as altered sensory feedback that would reasonably be expected to operate cross-modally. That this is fundamentally a motor phenomenon is further reinforced by the patients’ own perceptions of isolated, inexplicable acceleration in their behavior, over which they otherwise felt retained overall control. Continuous, simultaneous electroencephalography during the entire procedure showed no electrophysiological evidence of ictal activity, either during or after stimulation at other intracerebral electrodes.

Equally, this was not plausibly activation—always a possibility with electrical stimulation even if the overall effect is generally disruptive—of a specific movement pattern with a specific rate of alternation, for that would not explain why it was conditional on the action the patient was executing at the time, and why the stimulated pattern was always faster than baseline. A stimulated movement would be expected to be not only simple and monophasic, rather than complex, coordinated, and repetitive as here, but also morphologically identical, replacing the preceding action, rather than conditionally altering just one feature of it: its speed of execution. Repeated activation of a single movement component should have resulted in a tonic response, uninterrupted by its previously alternating rival, completely extinguishing the repetitive behavior in favor of just one component of it. Even if theoretically possible, at 50 Hz, the stimulation frequency was far too high to give rise to alternating movements of the observed frequency by resonance or interference with endogenous neural oscillations: that would in any rate have predicted a fixed resultant frequency across all stimulated conditions, not the increase from a self-determined baseline we observed. Finally, outwardly and as perceived by the patients themselves, this was morphologically essentially normal behavior, for it largely retained the features preceding its disruption, including the capacity to stop it altogether.

An intriguing alternative possibility, however, is that this is a manifestation of stochastic resonance within the human motor domain, a more complex phenomenon where non-linearities in the system cause the addition of noise not to degrade but to amplify the underlying neural signal [22]. Given that the effects here were observed at relatively high stimulation intensities, thought substantially to disrupt underlying activity, not just to add a degree of noise, this seems unlikely [18]. Note that a mechanism relying on a lower degree of disruption at a more distant locus where the intensity is lower, in “penumbra” fashion, is unlikely to explain our observations, for the penumbra will inevitably be larger than the point of stimulation, and so would likely have been comparably induced by stimulation of neighboring positions on the implanted grids. In any event, it is only within the context of a race model that enhancement of the activity of competing neuronal coalitions could plausibly result in a rate of behavior faster than the subject intends.

Disruptive stimulation of the present scale—a spheroid a few millimeters in diameter [23]—is not easily related to microstimulation in non-human primates, where the scale is finer, likely confined to a narrower subset of competing neuronal ensembles, and the currents lower, with potentially facilitatory effects. Procrastination here would thus be enhanced rather than reversed, resulting in delay, not acceleration. In the context of saccades, where saccadic direction may be taken as a marker of task specificity, facilitatory stimulation of the pre-supplementary motor area has been associated with relatively direction-insensitive increased latency, in broad agreement with our observations [6, 24, 25]. Nonetheless, the timing of stimulation in these studies strongly modulated the effect on latency, including reversing it when applied before a saccade was cued. In the closely related supplementary eye field, greater task specificity has been observed, including facilitated acceleration [25, 26, 27, 28], though it is interesting that controlled, memory-guided, highly contextual behaviors that more heavily engage medial structures have been more often delayed than accelerated.

Direct, interventional access to human cortex can only ever be justified clinically: the biological picture thereby framed is inevitably refracted through the lens of pathology. Although this sets a constraint on generalizing to wholly normal populations, we should note these patients were affected by a focal neurological disorder with predominantly intermittent functional manifestations that were absent during the study. Moreover, neither patient showed clinical evidence of any discernible impairments in their capacity for voluntary action.

Overall, disruption of a fundamental feature of decision-making, long predicted by race models of action, appears to be the most plausible explanation for the stimulation-induced phenomena in our patients. This observation reinforces the remarkable felicity of race models in understanding how we select our actions and urges the pursuit of their wider ramifications across the broader neural organization of voluntary action.

## Author Contributions

A.J. and B.D. contributed equally to the study as joint first authors. A.J., B.D., and P.N. conceived, conducted, analyzed, and reported the study. C.S. and A.W.M. contributed to the execution of the study in relation to behavioral testing and neurosurgical procedures, respectively.

## Supplemental Information

Document S1. Supplemental Experimental Procedures, Figures S1–S3, and Tables S1 and S2

Movie S1. Video and Audio Recordings of Behavior during Stimulation of the Critical Electrode Contacts, Related to Figure 2 The patient identifier and task are given before each video or audio segment. A red square indicates the timing of stimulation.

Document S2. Article plus Supplemental Information
