# Supplementary material for: Reversed Procrastination by Focal Disruption of Medial Frontal Cortex
Source: Curr Biol. 2016 Nov 7;26(21):2893–8. doi: 10.1016/j.cub.2016.08.016 (PMC5106371; doi:10.1016/j.cub.2016.08.016)
Supplement: Document S1. Supplemental Experimental Procedures, Figures S1–S3, and Tables S1 and S2 [file mmc1.pdf]

Current Biology, Volume *26*

Supplemental Information

Reversed Procrastination by Focal Disruption of Medial Frontal Cortex

Ashwani Jha, Beate Diehl, Catherine Scott, Andrew W. McEvoy, and Parashkev Nachev

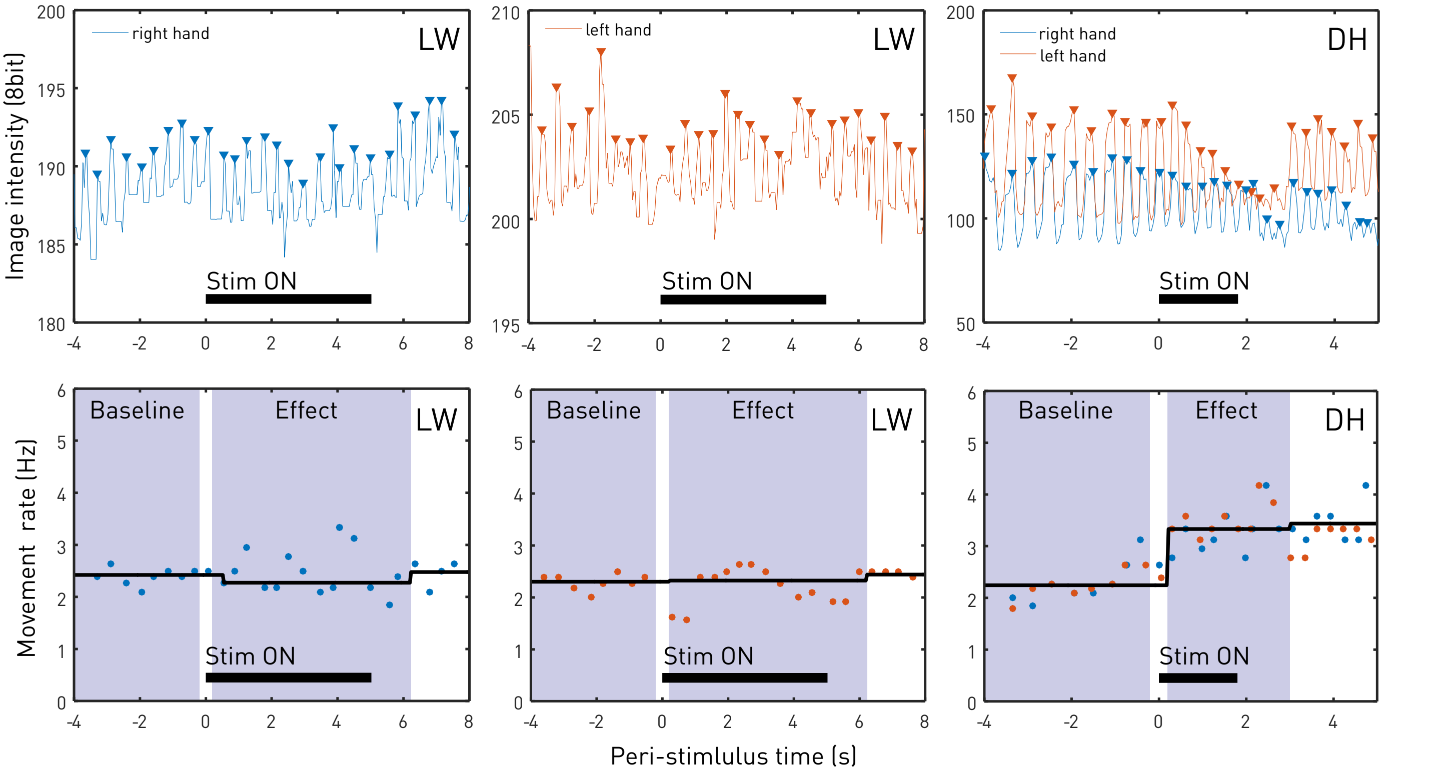

Figure S1. Parameterisation of manual movements during cortical stimulation. Related to Figures 2 and 3. The top row of plots shows the 8-bit greyscale image intensity values within the region of interest capturing the patient's fingers, for each hand, as a function of time, with the stimulation period marked in black above the abscissa. The triangles show automatically identified peaks. The bottom row shows the inter-peak intervals converted to instantaneous frequency by taking, for each point, the reciprocal of the average time interval between the preceding two movements. In grey are the windows defining the intervals included in the baseline and stimulation analyses. The black line is a 1st order spline with breakpoints at the window edges robustly fitted to the data for illustration. Note the clear change in manual task performance for DH, but not LW.

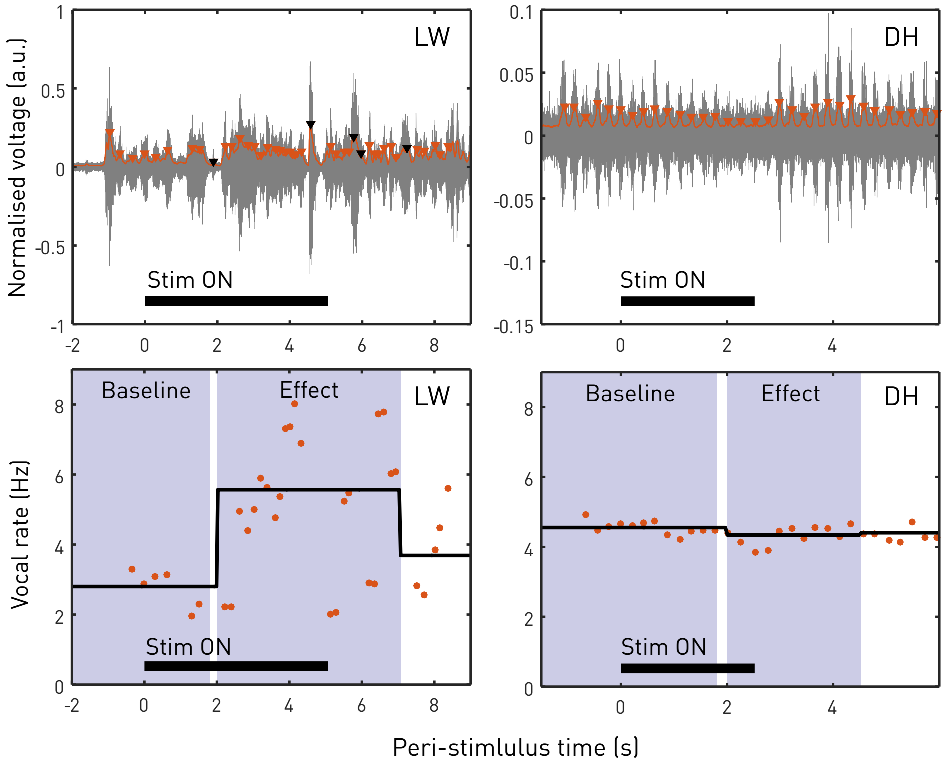

Figure S2. Parameterisation of vocal movements during cortical stimulation. Related to Figures 2 and 3. The top row of plots shows the high-pass filtered audio track voltage (in gray, normalised with 16 bit precision to arbirary units in the range -1:1), with the stimulation period marked in black above the abscissa. In red is the upper root mean square envelope of the signal, of which the red triangles show automatically identified peaks. Black triangles were manually removed artefacts. The bottom row shows the inter-peak intervals converted to instantaneous frequency as in the manual case, with 1st order splines identically fitted. Note the clear change in vocal task performance for LW, but not DH.

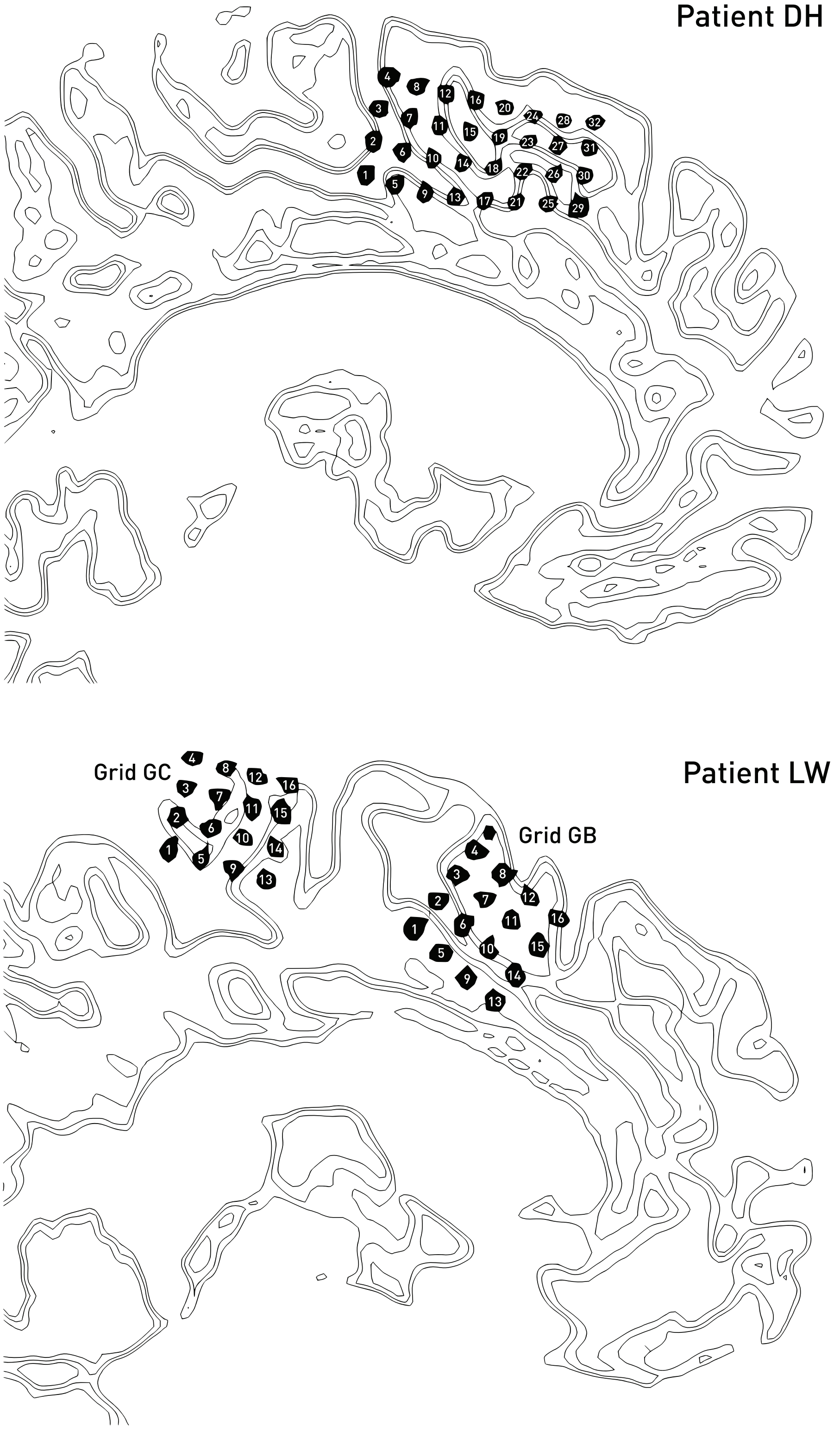

Figure S3. Electrode grid montages. Related to Figure 2. The labelling of electrodes in the relevant intracranial grids in participants DH (upper panel) and LW (lower panel) is shown. Stimulation effects at various bipolar electrode configurations within these grids are given in Table S2.

Table S1. Clinical details. Related to Figure 2.

Details	DH	LW
Age (at onset)	41 (29)	36 (12)
Handedness	R	R
Gender	M	F
Main seizure types	Atonic; tonic/clonic	Right arm/leg tonic; Atonic
Anti-epileptic medications	Lacosamide 100mg bd<br>Zonisamide 150mg bd	Tegretol Retard 700mg/800mg<br>Clobazam 10mg<br>Diazepam 5-10mg
Suspected zone	Left frontal	Left frontal
Language	Left dominant (fMRI)	Left dominant (fMRI)
Subdural grids (High density: 5 mm, centre-to-centre; regular density: 10 mm centre-to-centre	Medial wall:<br>High density: one 4x8, one 4x4.<br><br>Lateral wall:<br>8x8 frontoparietal,<br>2x8 anterior superior frontal gyrus,<br>1x6 middle frontal gyrus,<br>Two 1x6 inferior frontal gyrus.	Medial wall:<br>High density: one 4x4<br>1x6 strip.<br><br>Lateral wall:<br>8x8 frontoparietal
Stimulation currents during tasks	Manual (bilateral): 1mA<br>Vocal: 3mA	Manual (left): 3mA<br>Manual (right): 3.5mA<br>Vocal: 4mA

Table S2. Summary of stimulation effects (critical sites in grey). Related to Figure 2.

Subject	Contact pair		Task	Subjective effects	Stimulation parameters		Syllables or taps		Behavioural rate (Hz)			Statistics (*t*-test)		
					mA	*s*	*Pre*	*Post*	*Pre*	*Post*	*Δ*	*df*	*t*	*p*
LW	9	10	'lala'	None	4	5.06	7	24	2.77	5.14	2.37	27	-2.90	0.030
DH	9	10	'tata'	None	3	2.52	13	12	4.55	4.32	-0.23	21	2.42	0.980
LW	9	10	Tap (l)	None	3	5.03	10	16	2.34	2.33	-0.01	43	0.05	1.000
LW	9	10	Tap (r)	None	3.5	5.03	10	13						
DH	9	10	Tap (l&r)	None	1	1.62	17	19	2.27	3.39	1.12	32	-8.803	<0.001
DH	32	31	Reading	None	3	2.25	14	8	3.07	3.30	0.23	18	-0.54	0.60
DH	30	29	Reading	None	3	2.06	13	7	3.97	3.49	-0.48	16	0.65	0.53
DH	28	27	Reading	None	3	2.78	12	10	3.30	3.68	0.38	18	-0.63	0.54
DH	26	25	Reading	None	3	2.57	12	7	2.84	2.37	-0.47	15	1.09	0.29
DH	24	23	Reading	None	3	2.82	15	4	3.95	3.86	-0.09	15	0.11	0.92
DH	22	21	Reading	None	3	3.07	12	9	3.53	3.10	-0.43	17	0.66	0.52
DH	18	17	Reading	None	3	2.57	13	5	3.45	4.20	0.74	14	-0.79	0.44
DH	16	15	Reading	Speech	3	2.2	11	3	Too few syllables					
DH	14	13	Reading	None	3	2.81	11	11	3.39	3.63	0.25	18	-0.40	0.69
DH	12	11	Reading	None	3	2.65	13	8	3.27	2.73	-0.55	17	1.24	0.23
DH	8	7	Reading	Sensory	2	1.06	11	1	Too few syllables					
DH	6	5	Reading	Sensory	2	1.38	10	1	Too few syllables					
DH	4	3	Reading	None	1	2.7	8	8	3.11	2.63	-0.48	12	0.89	0.39
DH	2	1	Reading	None	3	3.12	8	7	2.13	2.36	0.23	11	-0.46	0.65
DH	3	2	Reading	Sensory	3	2.99	11	8	2.71	3.07	0.36	15	-0.48	0.64
DH	32	31	Tapping	None	3	2.78	3	20	Too few taps					
DH	30	29	Tapping	None	3	2.85	8	20	2.30	2.45	0.15	24	-1.28	0.21
DH	28	27	Tapping	None	3	2.62	1	17	Too few taps					
DH	26	25	Tapping	None	3	2.66	4	17	Too few taps					
DH	24	23	Tapping	None	3	1.66	16	1	Too few taps					
DH	22	21	Tapping	None	3	2.91	1	20	Too few taps					
DH	18	17	Tapping	None	3	2.27	3	17	Too few taps					
DH	14	13	Tapping	None	3	3.12	1	22	Too few taps					
DH	12	11	Tapping	None	3	3.35	1	24	Too few taps					
DH	8	7	Tapping	None	2	1.32	1	13	Too few taps					
DH	6	5	Tapping	None	3	0.96	3	10	Too few taps					
DH	3	2	Tapping	None	3	2.55	1	19	Too few taps					
LW-GC	4	8	At rest	Sensory foot	3									
LW-GC	12	8	At rest	Sensory foot	3									
LW-GC	12	16	At rest	Motor foot	3									
LW-GC	3	7	At rest	Motor foot	2									
LW-GC	11	7	At rest	Motor foot	2									
LW-GC	9	13	At rest	Sensory foot	2									
LW-GC	13	14	At rest	Motor arm	3									
LW-GC	14	15	At rest	Motor arm	3									
LW-GC	15	16	At rest	Motor arm	3									
LW-GC	10	11	At rest	Motor leg	2									
LW-GC	11	12	At rest	Sensory foot	2									
LW-GC	5	6	At rest	Sensory face	1									
LW-GC	6	7	At rest	Motor face	2									
LW-GC	7	8	At rest	Motor foot	2									
LW-GC	1	2	At rest	Motor foot	3	After-discharges								
LW-GC	2	3	At rest	Sensory foot	3									
LW-GC	3	4	At rest	Sensory foot	1									
LW-GB	13	14	At rest	Sensory foot	3									
LW-GB	15	16	At rest	Sensory foot	3.5									
LW-GB	14	15	At rest	Motor foot	3.5									
LW-GB	5	6	At rest	Motor arm	4									
LW-GB	6	7	At rest	Motor arm	4									
LW-GB	7	8	At rest	None	3									
LW-GB	1	2	At rest	Motor arm	3									

Supplemental Experimental Procedures

Participants

We studied two patients with non-lesional, medically-refractory, focal epilepsy during their clinical evaluation for potential neurosurgical treatment. The patients were admitted to the Sir Jules Thorn Telemetry Unit at the National Hospital for Neurology and Neurosurgery, London, UK. While no clear epileptic focus had been identified in either patient—hence requiring invasive neurophysiological study—the clinical picture and non-invasive neurophysiology included the left medial frontal wall within the range of possible targets. Both patients therefore underwent temporary implantation of subdural grids on the medial frontal cortical wall amongst other areas. The precise locations differed between the two patients for clinical reasons, but comparable coverage of the medial aspect of the superior frontal gyrus was obtained. The clinical details are given in Table S1.

The standard clinical protocol to formulate a hypothesis for seizure focus localisation includes the following: clinical assessment to identify seizure types and evolution of the epilepsy over time, scalp video telemetry to determine interictal and ictal source localisation of epileptic activity, structural and functional imaging including FDG-PET to determine presence of a lesion, functional MRI to determine language lateralisation, and neuropsychiatric and neuropsychological assessment to determine impact of the epilepsy and risk for resection. The decision whether or not a patient is a candidate for potentially curative resective surgery is made during a multidisciplinary team meeting, and whether or not intracranial EEG is first required for more precise focus localisation and/or mapping of eloquent cortex. A detailed plan for implantation of the intracranial electrodes is then devised. The intracranial neurophysiological evaluation relies on precise localisation of the electrode position by review of pre-implantation MRI fused to the post-implantation CT. Continuous video EEG telemetry identifies intracranial epileptform activity both between and during seizures. Further, direct electrical cortical stimulation of each implanted electrode is performed to map eloquent cortex and to predict the behavioural consequences should the region be resected.

Below we set out the aspects of the protocol relevant to our study.

Informed consent for research analysis of the clinical data was obtained prior to testing following our standard clinical guidelines. The patient's identifying initials here bear no relation to their own, and all plausibly identifying information has been removed.

Clinical details

LW: At the time of evaluation, LW was a 36 year-old, right-handed woman with frequent (>10 a day) disabling seizures dating from the age 12, characterised by right leg and arm tonic seizures that occasionally generalised, as well as rarer atonic seizures, including complete inability to move with preserved consciousness. Scalp EEG supported a left frontal or

frontocentral focus with left frontocentral interictal spikes; seizures showed a left frontocentral ictal pattern. The neurological examination was unremarkable. Neuropsychometric assessment did not reveal any significant cognitive deficits. An FDG-PET scan showed no areas of significant brain hypometabolism.

DH: At the time of evaluation, DH was a 41 year-old right-handed man with atonic seizures and generalised tonic clonic seizures dating from the age of 29. Scalp EEG had revealed slowing, but no spikes, over the left frontocentral region. Numerous seizures with atonia and right head version followed by a right arm clonic seizure were also recorded, and ictal EEG implicated the medial frontal region. Neurological examination was normal. Neurocognitive assessment showed mild deficits supportive of dominant frontal lobe involvement. An FDG-PET scan was unremarkable.

Language fMRI studies revealed left language dominance in both patients.

Structural imaging

Data acquisition

The same protocol was followed in both patients. Pre-operatively, a whole-brain, T1-weighted, magnetic resonance imaging of resolution 0.94x0.94x1.1mm resolution was acquired on a 3T General Electric Excite HDx scanner (General Electric, Milwaukee, WI, USA) using an eight-channel array head coil for reception and the body coil for transmission, with standard imaging gradients (maximum strength 40 mT/m and slew rate 150 T/m/s). Post-electrode implantation, the patients underwent an uncontrasted, whole-head, CT scan of resolution 0.43x0.43x1.2mm. (SOMATOM Definition 128-slice, Siemens Healthcare GmbH, Erlangen, Federal Republic of Germany). LW's neurophysiological study was carried out 4 days after the post-implantation CT scan. Since DH's neurophysiological study was carried out 4 weeks after the initial CT scan, we used instead a later scan of the same parameters, carried out within 3 days of stimulation.

Analysis

We sought to determine as precisely as the data allowed the location of the stimulation sites, both in relation to the patient's individual cortical anatomy, and in standard stereotactic space. This required two forms of image registration: a native space, within-subject registration of the post-implantation CT to the pre-implantation MRI, and a MNI space, template registration of the MRI, applying the derived parameters so as secondarily to warp the natively registered CT into the same space.

CT-MRI registration

Ordinarily, within-subject registration of one imaging modality against another requires only an affine transform, for the underlying morphology of the brain is the same. Where a craniotomy has been performed, however, a degree of non-linear adjustment is necessary to

compensate for the minor but significant distortions opening the skull introduces. This is challenging in the context of post-implantation CT because of the relative poverty of tissue contrast and the presence of electrode artefact. Nonetheless, since the routine post-operative dural collections principally responsible for the distortion cause very smooth deformations, it is possible to introduce sufficient constraint into the non-linear step to ensure the transformation remains biologically plausible. Here we develop a novel approach to performing non-linear CT-MRI registration, and validate it in our two patients with the aid of independently identified anatomical landmarks. Unless otherwise indicated, all operations were performed within SPM12 (http://www.fil.ion.ucl.ac.uk/spm/), running in Matlab R13b (http://uk.mathworks.com/), on a 64-bit machine under Windows 7 SP1. The procedure was identical for each patient.

CT PRE-PROCESSING: First, a rigid body coregistration to the standard SPM12 tissue probability map was performed based on normalised mutual information with adjustment from a Procrustes analysis weighted by the white and grey matter compartments. This placed the scan in rigid register with the MNI template space. So as to focus subsequent processing on tissue-relevant contrast, an identical copy of the scan was windowed so as to zero all voxels outside the range of 0 to 100 Hounsfield units. This was then filtered with an Oracle-based 3D discrete cosine transform filter[S1] to enhance tissue contrast. All subsequent operations were performed on this image, and the final transformation was replicated on the original image at the end.

MR PRE-PROCESSING: As for the CT, a rigid body coregistration to the standard SPM12 tissue probability map was performed based on normalised mutual information with adjustment from a Procrustes analysis weighted by the white and grey matter compartments. This placed the scan in rigid register with the MNI template space, and also with the CT. The scan was then resliced using 4th degree *b*-spline interpolation to the same bounding box and voxel size as the CT. SPM12's standard unified segmentation and normalisation procedure, with default parameters, was used to generate segmented compartments in native space for each of the standard 6 tissue classes, as well as a set of parameters for non-linear transformation into MNI space of this and any other image in register with it.

LANDMARK ANNOTATION: A set of 73 anatomical landmarks was manually and independently applied to each scan by a trained neurologist (PN) with the aid of MIPAV's triplanar visualisation and volume-of-interest tools (http://mipav.cit.nih.gov/). In common with established practice[S2], the landmarks fell into characteristic planes established by the anterior commissure (AC) – posterior commissure (PC) alignment achieved in the initial rigid body registration to MNI space, as follows:

Axial plane of the corpus callosum (CC) (separately for each hemisphere): lateral ventricle anterior, caudate nucleus anterior, caudate nucleus posterior, putamen anterior, putamen medial, putamen posterior, insula anterior, insula posterior, and lateral ventricle posterior.

Coronal plane through AC (separately for each hemisphere except for AC itself): lateral ventricle superior, lateral ventricle inferior, caudate superior, putamen superior, putamen inferior, globus pallidus medial, insula superior, and insula inferior.

Mid-sagittal plane: anterior cerebrum (AC-PC plane), paracingulate gyrus anterior (AC-PC plane), cingulate gyrus anterior (AC-PC plane), cerebrum posterior (AC-PC plane), corpus callosum anterior, corpus callosum posterior, corpus callosum genu angle, corpus callosum tip of genu, corpus callosum splenium centre, corpus callosum splenium posterior tip, anterior cerebrum (CC plane), paracingulate gyrus anterior (CC plane), cingulate gyrus anterior (CC plane), cerebrum posterior (CC plane), thalamus centre, cerebrum superior (AC coronal plane), corpus callosum superior (AC coronal plane), corpus callosum inferior (AC coronal plane), corpus callosum superior (PC coronal plane), corpus callosum inferior (PC coronal plane), calcarine fissure-parietooccipital fissure, parietooccipital fissure-hemisphere margin, calcarine fissure-hemisphere margin.

Lateral parasagittal planes (through insula, separately for left and right): anterior horizontal ramus of Sylvian fissure-Sylvian fissure, anterior ascendant ramus of Sylvian fissure-Sylvian fissure, inferior frontal fissure-hemisphere margin, precentral fissure-hemisphere margin, central fissure-hemisphere margin, postcentral fissure-hemisphere margin, Sylvian fissure posterior, Jensen's fissure-hemisphere margin.

Each set of landmarks was used to generate a volume image in register with, and of the same dimensions as, its corresponding scan. Any transformation applied to the scan could then be identically applied to the landmark image, allowing direct, unbiased quantification of the fidelity of registration. Registration error was indexed as the root mean squared distance (and its standard deviation) across all landmarks, taking the MR scan as the reference and each transformed CT scan as the test. The baseline registration error—the difference between the sets of landmarks following the initial rigid registration—was 3.12 mm (std=1.63 mm) for DH and 4.68 mm (std=2.07 mm) for LW.

NON-LINEAR REGISTRATION OF CT TO MR: The relatively small mean error—only a few millimetres—suggests only relatively minor non-linear adjustment is necessary. Here we performed this adjustment by applying SPM12's unified segmentation and normalisation procedure on the windowed, filtered CT scan, but instead of using the standard MNI space template tissue compartments as tissue prior probability maps we used the individual MR-derived tissue compartments in *native* space. The resultant transformation is therefore not into standard stereotactic space but rather the native space of the T1 (which the CT already shares), adjusted to introduce some conformity with the native MR tissue segmentation. Other than removing the affine registration step and any bias correction, the parameters of the algorithm were otherwise as default in SPM12. Note that since SPM12's routine involves explicitly modelling anomalous signal, this adjustment was robust to the artefact created by the metal grids. Inspection of the tissue compartments from the CT segmentation showed that the critical grey and white matter partitions were not significantly contaminated by the artefact, though of course it did limit the extent to which the neighbouring tissues could

materially contribute to the deformation. So as to optimise the degree of laxity in the transformation, the procedure was repeated with different levels of smoothing applied to the MR tissue compartments before they were used as priors, in the range of a Gaussian kernel of 1 to 8 mm full width half maximum, in 1 mm steps. The deformation field describing the transformation was applied both to the CT and its corresponding landmark image. Tested against the MR, the smallest landmark error was found with a kernel of 4mm width: this was therefore used in the final analysis. The final root mean square errors were 3.09 mm (std=1.35 mm) for DH, and 3.88 (std=2.32 mm) for LW.

Note that the registration process itself *does not use the landmarks in any way.* Optimising the transformation by refining the degree of smoothing of the priors is therefore not in any danger of creating an artificial overfit to the data, as the landmark proximity reflects—but does not drive—the process.

NORMALISATION (TRANSFORMATION INTO MNI SPACE): The deformation field estimated from the MR scan segmentation and normalisation was applied to the grey matter compartment of the MR image, producing a probabilistic map of grey matter transformed in standard MNI space. Since the MR was a standard high-resolution, volumetric, unlesioned scan, there were no grounds to suspect the quality of registration, and no need for landmark analysis of this step. The same deformation field was used to transform the final MR-registered CT into the same space. Both transformed images were resliced to 1mm isotropic voxels. The output of this stage was thus two MNI transformed maps: one of MR-derived, grey matter, and another of the CT-derived electrode locations.

Visualisation

So as to visualise the location of the electrodes, the normalised images were rendered in ParaView (http://www.paraview.org/) as thresholded 3D contours. For the grey matter image, three contours were created for tissue probability values of 0.9, 0.8, and 0.7. A parasagittal slice cutting through the brain at x = -4 thus created a set of isolines as a probabilistic guide to the sulcal boundaries of the left medial wall in that plane. The displayed region of interest was confined to the coordinates comfortably capturing the frontal lobe. To visualise the grid contacts, the CT contour was thresholded at the Hounsfield value that optimally identified them visually, and the displayed region of interest was confined to display the stimulated grids in the target region of the medial wall. The *y* and *z* coordinates for the region of interest were plotted using ParaView's native axis display function.

Functional imaging

Data acquisition

The same protocol was followed in both patients. Functional MRI data were acquired as gradient-echo planar T2*-weighted images providing blood oxygenation level–dependent (BOLD) contrast. Echo time (TE) was 30 ms and repetition time (TR) 4.5 s. Each volume

comprised 58 contiguous 2.5 mm oblique axial slices, through the temporal and frontal lobes with a 24cm field of view, 96 x 96 matrix, reconstructed to 128 x 128 for an in-plane resolution of 1.88 x 1.88mm. The field of view was positioned to maximize coverage of the frontal and temporal lobes. The data were acquired on 3T General Electric Excite HDx scanner (General Electric, Milwaukee, WI, USA). All functional imaging was done before implantation.

The task consisted of eight blocks lasting 30 seconds each, interspersed with periods of rest fixation of the same length, where the patients were visually presented with concrete nouns every 3s, and were asked either covertly to generate verbs associated with these nouns (indicated by the letter ''G'' preceding the noun), or silently to repeat the nouns presented (indicated by the letter ''R'' preceding the noun). Each block contained trials of the same type, with the verb generation and noun repetition alternating across blocks.

Analysis

Imaging data were analysed using SPM12 (http://www.fil.ion.ucl.ac.uk/spm/). The imaging time-series of each subject was realigned using the mean image as a reference, and smoothed with a Gaussian kernel of 8 mm full-width half maximum (FWHM). At each voxel, the time-series BOLD data were entered into a general linear model with the conditions modelled as a box-car function regressors convolved with a standard haemodynamic response function. The realignment parameters were added as nuisance regressors to minimize artefactual activation owing to head movement correlated with the task. The main effect of task was estimated separately for each condition. The SPM(t) images were thresholded at p<0.05 FWE corrected, except in the noun repetition condition for DH were the level of activation necessitated the lower threshold of p<0.001. So as to bring the statistical maps into MNI space, the mean image was rigidly co-registered to the preimplantation structural MR volume, and the deformation field from the structural MR normalisation described in the preceding section was applied to each SPM(t) map.

Visualisation

So as to visualise the areas of significant activation on the medial wall, the normalised SPM(t) maps were rendered in ParaView (http://www.paraview.org/) as thresholded 3D contours, with the thresholds set as described above. The displayed region of interest was confined to the medial frontal wall, extending into the left hemisphere as far as x = -4 and into the right as far as its medial surface. Noun repetition and verb generation are expected to differ in the relative balance of supplementary motor area and presupplementary motor area involvement: rather than show the explicit contrast between them here we plotted both in different colours, semi-transparently rendered so as to display their overlap.

Electrode grid implantation and stimulation

A standard clinical approach was adopted in each patient. In brief, the previously described structural MR imaging was used to plan the implantation of subdural grid and strip

electrodes (AD-TECH R Medical Instrument Corporation), which was performed through a frontoparietal craniotomy. In both patients, high-density electrode grids (5mm centre-to-centre inter-contact spacing) were used for left mesial frontal cortex coverage, whilst larger 8x8 grids (10mm centre-to-centre inter-contact spacing) and electrode strips were used to cover other regions (see Figure S3 and Table S2). Electrode localisation was confirmed via intra-operative photographs, and by post-operative CT as described above.

Post-operative cortical stimulation was performed using bipolar stimulation techniques[S3]. To avoid stimulation induced seizures, antiepileptic medication was reintroduced prior to cortical stimulation mapping (see Table S1). Trains of 50-Hz, bi-phasic square wave pulses of an AC-current with a pulse width of 500microseconds were delivered by a Nicolet™ cortical stimulator used with C64-OR amplifiers and a Nicolet Cortical stimulator Control unit (ISO 13485, ISO 9001; Nicolet Biomedical, Madison, US). Current intensity was gradually increased from 1mA in increments of 0.5 or 1mA up to 7.5mA (15 mA peak to peak of the biphasic stimulus), until the occurrence of a clinically obvious change or after-discharges on EEG monitoring. The interval between increments varied depending on the presence or absence of either behavioural or neurophysiological manifestations but was generally no shorter than 30 seconds. In line with clinical practice, so as to detect any task-invariant behavioural effects, stimulation was initially performed at rest: where incrementing to 3 to 4 mA could be achieved without visible or patient-reported motor or sensory phenomena, task-specific performance was subsequently evaluated. This clinical requirement inevitably constrained the number of sites where task-specific effects could be explored: only where no overt behaviour is triggered by stimulation can the effect on a task be fruitfully examined.

The stimulation currents for the specific tasks and electrodes reported here are presented in Table S2. Intracranial EEG data were simultaneously recorded throughout stimulation on a 128-channel NicoletOne™ LTM system (Nicolet Biomedical, Madison, US) sampling at 512 Hz.

Behavioural tasks

Data acquisition

The behavioural evaluation followed standard clinical practice, which is deliberately flexible so as to adapt to the patient's specific requirements and abilities. All testing was carried out in the patient's telemetry room, during continuous video, audio, and neurophysiological monitoring, with two neurological clinicians and a neurophysiologist present. As outlined above, stimulation began at low currents, with the patient at rest, escalation both to higher currents and during behavioural tasks only where the absence of overt behavioural effects—either reported by the patient or observed by the clinician—made this possible.

The manual task consisted of rhythmic flexion-extension movements at the metacarpophalangeal joints of each hand, either unilaterally or both hands together, and

either one finger or all at the same time. The vocal task consisted of repeated utterance of a single syllable such as "la", "ma", or "ta", or reading aloud of a fixed paragraph of text. For each task, the desired movement was first demonstrated to the patient, then he or she performed it unprompted. Although a rate of two or three Hz was implicitly suggested in the demonstration, the patient was free to choose whatever rate felt most comfortable, but was encouraged to keep it constant. No temporal entraining of any kind was applied. The patient was instructed to continue the behaviour until either a request from the clinician to stop or a perceived physical change of any kind: sensory, motor, or cognitive. Stimulation was applied during this interval, at a manually randomised onset in relation to the beginning of the behaviour, and continued for a few seconds or until a behavioural change was observed or reported by the patient. Although the patient naturally knew a stimulation would be performed at some point during the action, no warning of the specific point of onset was given. Where no behavioural effect was observed, the stimulation intensity was escalated as described above until either a response was elicited or the maximum threshold was reached. Where the effect was equivocal, clinically, the stimulation was repeated once with the same parameters. The same behavioural tasks were as far as possible tested for each adjacent pair of electrode locations. The presence of after-discharges limited testing at a small minority of sites; no after-discharges were seen at our critical sites.

In our two patients, during stimulation at the critical electrodes, the manual task performed by DH was bimanual, and involved all fingers; in LW it was unimanual and involved the index finger. The vocal task in DH involved the syllable 'ta'; in LW the syllable 'la'. The video and/or audio record for both tasks when the critical electrodes were stimulated is available in the supplemental video material. Table S2 provides a summary of the behavioural parameters together with details of the post-hoc statistical tests based on the mixed general linear model quoted in the main text.

The accelerated manual behaviour seen in DH and vocal behaviour seen in LW was captured after stimulation of a unique electrode pair in each case. To investigate the anatomical specificity of the effects, we also present in Table S2 the results of stimulation at neighbouring electrodes, obtained across a range of tasks.

Note that cortical stimulation is carried out for clinical purposes, within a highly individualised context that introduces inevitable variations between patients, in addition to those resulting from different electrode placement. Crucially, the ability to perform a task during stimulation was conditional on there being no overt behavioural response that would otherwise interfere with performance. This included both motor and sensory phenomena. After-discharges and overt epileptiform neurophysiological phenomena also limited testing at some sites.

The relatively rostral localisation of the majority of DH's electrode contacts—where overt behavioural effects are rarely observed—explains the large proportion of sites open to investigation of task performance. Where enough data was available to allow a quantitative assessment (a *t*-test comparing pre- and post-stimulation rate; see Supplemental Methods),

no significant effects were found (see Table S2). Other stimulation effects in the non-critical electrodes—such as sensory phenomena—were largely restricted to electrodes located on the posterior edge of the grid. Note that conditions where there were too few syllables or taps (<4) were not quantitatively analysed. In this *post hoc* analysis, all *p* values are greater than 0.05 uncorrected (shown), and therefore further Bonferroni adjustment will not affect the inference.

The scope of task-related stimulation trials was limited in LW by overt sensory and movement phenomena that precluded the performance of any task (see Table S2).

Data analysis: movement parameterisation

The patient's motor behaviour was parameterised and quantified from the telemetry video and audio data using custom scripts written in Matlab (The MathWorks, Inc., Natick, MA). In brief, the timing of each finger movement was determined by extracting a time-series of greyscale image intensity values (at the original video sample rate of 25 frames per second) within a manually defined region-of-interest enclosing the fingers. This produced an oscillating trace where the peaks corresponded to each cycle of movement; this was manually checked in each case (Figure S1). For the purposes of detecting a stimulation-induced change, the frequency of alternation was then calculated as the reciprocal of the mean time delay between the neighbouring (preceding two) movements. For the reciprobit analysis (see below) each inter-movement interval was analysed independently.

Vocal data were extracted from the audio channel of the video telemetry record (16 bit, sampled at 11kHz) and high pass filtered at 300Hz using a Finite Impulse Response filter implemented in Fieldtrip (http://www.ru.nl/neuroimaging/fieldtrip/)[S4] to remove background noise. The upper Root Mean Square envelope of this signal provided a 'volume envelope' time-series of speech, each peak identifying a syllable. Matlab's peak detection algorithm was then used to determine the timing of spoken syllables from this signal (Supplemental Figure S2). As in the manual task, the parameterisation was verified by inspection, and non-speech sounds (such as breaths) removed before conversion to behavioural frequency as above.

Data analysis: inference

The behavioural effect of stimulation at the critical locus was clinically apparent. To formalise the inference, we entered baseline and stimulation frequency values into a mixed general linear model, with subject as a random factor and effector modality (vocal versus manual) and stimulation (pre or post) as fixed factors. Post hoc *t*-tests were used to interrogate significant effects. To account for multiple comparisons, we performed a Bonferroni adjustment to the critical threshold of alpha=0.05.

The baseline and stimulation periods included in the models were defined separately for each condition and designed to be cognizant of the effector-specific effects of stimulation, controlling for as many parameters as is possible in this clinical scenario, where stimulation

lengths and block lengths varied. Note that since the timing of the clinical manifestations relative to the onset and offset of stimulation is substantially variable, and differs across effectors—generally showing greater latency for speech than for manual movements[S5]—there is neither a hard criterion for setting the correct lag nor plausible grounds for keeping it the same across effectors. In keeping with the clinically observed effector-specific differences, for the manual task we therefore set the lag relatively short, at 200ms, comparing a 4 second baseline window to a stimulation window beginning 200ms after the onset of stimulation and extending for a further 1.2 seconds beyond the offset of stimulation. For the vocal task, where a slower onset is to be expected, the stimulation window was set 2 seconds after the onset of the stimulation, continuing for 2 seconds after offset. The correspondence with the data is illustrated by a first order spline, fit using a robust minimisation of ordinary least squares with breakpoints at the baseline and stimulation window edges (implemented in the SplineFit Toolbox http://uk.mathworks.com/matlabcentral /fileexchange/13812-splinefit) and shown in the bottom panels of Figures S1 & S2.

Where the vocal task consisted of reading, the syllable separation is naturally reliant on the sensitivity of the volume envelope to neighbouring phonemic differences, which are also likely to be less pronounced within words than between them. This effect can be viewed as modest downsampling of the data (because some syllables will be missed). It cannot plausibly create spurious components, nor plausibly interact with stimulation in a way that may confound the result.

To make our analysis sufficiently robust, we rejected behavioural trials where the total number of syllables or finger movements recorded was too low for a statistical analysis to be meaningful ($n<4$), in either the pre or post-stimulation conditions.

LATER modelling

To explore the correspondence of the data to a LATER model of action here, we transformed the inter-movement intervals, for each of the two conditions where a significant stimulation effect was observed, into their reciprocals and plotted them against their *z*-scores, assuming them to be Gaussian in their distribution after the transformation. An overview of the underlying LATER model is given in Figure 1, and of such "reciprobit" plots in Figure 3 of the main text.

The data from each condition were separately fitted to the output of LATER models of the early (where present) and main components of the transformed distributions. In the pre-stimulation data—defining the default behaviour—the main component was modelled as a ramp process racing at rate r to a threshold of fixed height $\theta=1$ above baseline. Three parameters are allowed to vary: μ, σ_1, and σ_2. The rate of rise, r, is a random Gaussian variable of mean, μ, and standard deviation, σ_1. The early component process rises at a rate set at '0', with much larger standard deviation, σ_2. To determine the effect of stimulation, four different models of the post-stimulation data were separately fitted for each condition (manual and vocal), where the three standard parameters were constrained to different extents. The "Null" model assumes no effect of stimulation, and fits both pre- and post-

stimulation data with the same parameters. The "Unconstrained" model, treats the pre- and post-stimulation data as unrelated, allowing all three parameters to vary freely. The "Swivel" model is identical to the "Null" model except that the θ parameter is no longer fixed at 1, and is allowed to change with stimulation. The "Shift" model is identical to the "Null" model except that the μ parameter is allowed to change with stimulation.

These models correspond to materially different physiological effects of stimulation on the underlying behaviour, as conceptualised within LATER. A "Swivel", where stimulation produces a rotation of the modelled distribution around an intercept at infinity, implies a change in the threshold that a process must reach first to become manifest in behaviour. A "Shift", where stimulation produces a horizontal displacement of the modelled distribution along the abscissa, implies a change in the rate of rise of the competing processes. The other two—"Null" and "Unconstrained"—model either the absence of any stimulation effect, or its indifference to the distinction that shift and swivel define.

All models were estimated by minimising their negative log likelihood, which was then summed across subjects to provide a cumulative log likelihood per model. To compare the models, we used the Bayesian Information Criterion (BIC), which optimally balances differences in log likelihood taking into consideration model complexity (the number of model parameters that can vary) and number of data samples. We also supply a categorial interpretation of the strength of the evidence provided, based on BIC and its relation to the Bayes Factor (e.g. change in BIC >~2.2 is substantial positive evidence, change in BIC>~6 is very strong evidence) [S6]. The comparison of models allows inferences to be made on whether the effect of stimulation is most parsimoniously explained by a change in distance to threshold, θ ('Swivel'), mean rate of rise μ ('Shift'), or neither alone ('Unconstrained'), as compared to the 'Null' model.

In a further, corroborative analysis, we extracted the maximum likelihood value of the parameters of interest from the winning model, and calculated their confidence intervals with the aid of a bootstrap procedure. Data from the pre- and post-stimulation conditions (separately for manual and vocal responses) were randomly resampled with replacement to create an artificial dataset of the same size as the original, from which the model could be repeatedly re-estimated. This procedure was performed 1000 times and the 2.5% and 97.5% centiles of the resulting parameter estimates were used to approximate the 95% confidence intervals[S7].

These analyses were performed with the aid of Mike Shadlen's Reciprobit Toolbox v1.0 for Matlab (https://www.shadlenlab.columbia.edu/), in addition to custom Matlab scripts available from the authors.

Supplemental References

S1. Manjón, J.V., Coupé, P., Buades, A., Louis Collins, D., and Robles, M. (2012). New methods for MRI denoising based on sparseness and self-similarity. Medical Image Analysis *16*, 18–27.

S2. Grachev, I.D., Berdichevsky, D., Rauch, S.L., Heckers, S., Kennedy, D.N., Caviness, V.S., and Alpert, N.M. (1999). A method for assessing the accuracy of intersubject registration of the human brain using anatomic landmarks. Neuroimage *9*, 250–268.

S3. Kovac, S., Scott, C.A., Maglajlija, V., Toms, N., Rodionov, R., Miserocchi, A., McEvoy, A.W., and Diehl, B. (2014). Comparison of bipolar versus monopolar extraoperative electrical cortical stimulation mapping in patients with focal epilepsy. Clinical Neurophysiology *125*, 667–674.

S4. Oostenveld, R., Fries, P., Maris, E., and Schoffelen, J.-M. (2010). FieldTrip: Open Source Software for Advanced Analysis of MEG, EEG, and Invasive Electrophysiological Data. Computational Intelligence and Neuroscience *2011*, e156869.

S5. Kovac, S., Scott, C.A., Maglajlija, V., Rodionov, R., McEvoy, A.W., and Diehl, B. (2011). Extraoperative Electrical Cortical Stimulation: Characteristics of Motor Responses and Correlation with Precentral Gyrus. Journal of Clinical Neurophysiology *28*, 618–624.

S6. Kass, R.E., and Raftery, A.E. (1995). Bayes Factors. Journal of the American Statistical Association *90*, 773–795.

S7. Efron, B., and Tibshirani, R. (1986). Bootstrap Methods for Standard Errors, Confidence Intervals, and Other Measures of Statistical Accuracy. Statist. Sci. *1*, 54–75.
