# Supplementary figures and images for: Reversed Procrastination by Focal Disruption of Medial Frontal Cortex
Source: Curr Biol. 2016 Nov 7;26(21):2893–8. doi: 10.1016/j.cub.2016.08.016 (PMC5106371; doi:10.1016/j.cub.2016.08.016)

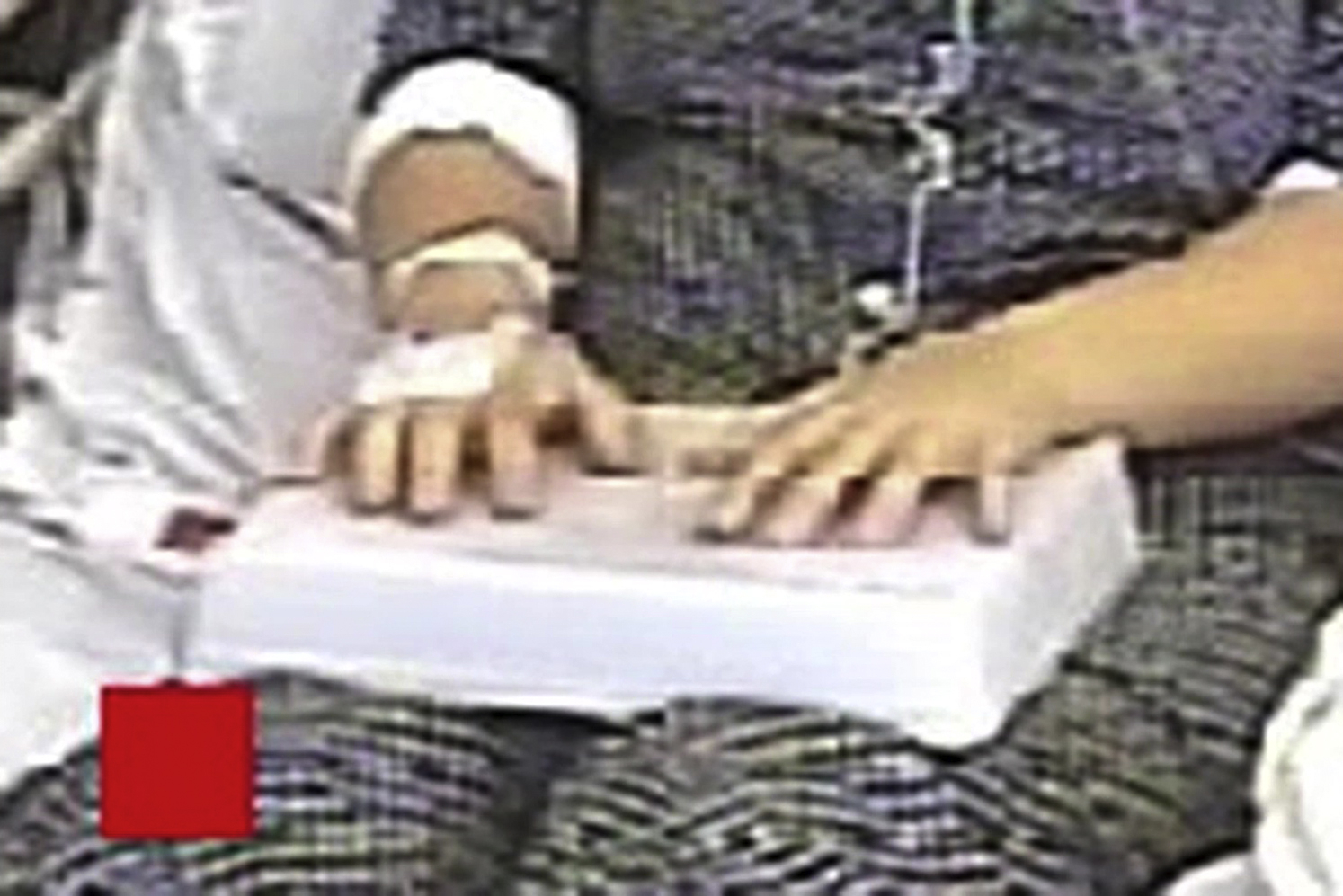

Supplement: Movie S1. Video and Audio Recordings of Behavior during Stimulation of the Critical Electrode Contacts, Related to Figure 2 — The patient identifier and task are given before each video or audio segment. A red square indicates the timing of stimulation. [file mmc2.jpg]
